# Editorial: Advances in wildlife parasitology and host-pathogen dynamics

**DOI:** 10.3389/fvets.2026.1933958

**Published:** 2026-09-02

**Authors:** Miljenko Bujanić, Jovan Mirčeta, Relja Beck

**Affiliations:** 1 Faculty of Veterinary Medicine, University of Zagreb, Zagreb, Croatia; 2 Directorate for Food Safety, Veterinary and Phytosanitary Affairs, Podgorica, Montenegro; 3 Croatian Veterinary Institute, Zagreb, Croatia

**Keywords:** ecological dynamics, environmental impact on disease dynamics, host-pathogen interactions, parasite life cycles, wildlife parasitology, zoonotic diseases

Wildlife parasitology is an essential field for understanding the complex interactions between wildlife hosts, parasites, and the environments they share. The importance of these interactions extends beyond parasite biodiversity and wildlife health, as changes in host–parasite relationships may also have implications for domestic animals and human populations. The common perception of parasites as inherently harmful represents an oversimplified view of far more complex and dynamic biological interactions. With the exception of non-native parasite species, which may cause substantial damage when introduced into immunologically naïve hosts and can even contribute to local population declines or extinctions (1, 2), most parasites have evolved in close interaction with their hosts and often coexist with them without causing severe disease (3).

Host–parasite relationships, however, are not static. They continuously change through ecological and evolutionary interactions between parasites, hosts, and their environments. A recent example is the non-native trematode *Fascioloides magna* in Europe, which appears to be developing a more balanced interaction with roe deer (*Capreolus capreolus*), previously considered an aberrant host in which infection was frequently fatal (4). Such observations illustrate how host–parasite interactions may change over time and emphasize the importance of long-term wildlife studies. At the same time, habitat modification, climate change, host population expansion, and biological invasions create new opportunities for contact between parasites and potential hosts, reshaping established transmission cycles.

The contributions included in this Research Topic illustrate these interactions from different ecological, epidemiological, and methodological perspectives. Together, they demonstrate that wildlife parasitology is increasingly moving beyond simple records of parasite occurrence toward a broader understanding of host associations, transmission pathways, environmental influences, and evolutionary change.

Šikić et al. provided the first molecularly confirmed identification of *Spirometra mansoni* in golden jackals (*Canis aureus*) in Croatia and possibly the first such report from this host in Europe. This finding expands the known host and geographical range of *S. mansoni* and illustrates how molecular methods may reveal parasite–host interactions that have previously remained undetected or taxonomically unresolved. The occurrence of this cestode in an expanding mesocarnivore such as the golden jackal also raises questions regarding the role of wildlife hosts in maintaining and dispersing *Spirometra* spp. across European ecosystems.

Another unusual host–pathogen interaction was described by Mihalca et al., who reported erythema migrans-like lesions in a European badger (*Meles meles*) from Romania. Histopathological examination revealed mild chronic inflammatory changes consistent with such lesions, while molecular analysis confirmed *Borrelia afzelii* DNA in the affected skin. Erythema migrans is primarily recognized as a characteristic clinical manifestation of Lyme borreliosis in humans, making its observation in a wild mammal particularly interesting. The study suggests that wildlife hosts may exhibit clinical or pathological responses to *Borrelia burgdorferi sensu lato* that remain poorly recognized. It also highlights the importance of studying not only the presence of pathogens in wildlife but also their interactions with hosts and the biological consequences of infection.

The interaction between wild and domestic transmission cycles was addressed by Nagy et al., who investigated *Dirofilaria immitis* infection in golden jackals (*Canis aureus*), red foxes (*Vulpes vulpes*), and Eurasian badgers (*Meles meles*) along the northern edge of the Mediterranean climatic zone. Despite apparently suitable environmental conditions, infection prevalence was low, reaching 2.3% in jackals and 1.4% in foxes, while no infections were detected in badgers. Temperature and socioeconomic factors influenced the spatial distribution of infection, whereas precipitation and land use showed no detectable effect. These findings suggest that wild mesocarnivores in the studied area may primarily reflect spillover from domestic transmission cycles rather than acting as major reservoirs of canine heartworm. This study clearly demonstrates that the presence of suitable hosts and environmental conditions does not necessarily result in an equally important epidemiological role for all host species.

The continuing spread and changing host interactions of *Fascioloides magna* in Europe were further illustrated by Žele Vengušt et al. The authors molecularly confirmed the parasite in red deer (*Cervus elaphus*), roe deer (*Capreolus capreolus*), and fallow deer (*Dama dama*) in Slovenia. Together with previous observations of changing parasite–host interactions, these findings demonstrate the ability of an introduced parasite to persist, spread, and interact with multiple European cervid species. The European history of *F. magna* provides an excellent example of how parasite invasion is followed by continuous ecological and evolutionary interactions with newly encountered host populations.

While *F. magna* represents an example of a parasite introduced beyond its native range, biological invasions may also involve the movement of hosts carrying their own parasites. Such host introductions can establish entirely new parasite–host interactions and, in some cases, introduce zoonotic parasites into previously unaffected environments. Benovics et al. reported the first confirmed occurrence of *Baylisascaris procyonis* in free-ranging invasive Northern raccoons (*Procyon lotor*) in the Czech Republic. The finding expands the known distribution of this zoonotic nematode in Central Europe and highlights the epidemiological consequences of invasive host expansion. As raccoon populations spread, parasite eggs may accumulate in the environment, creating new opportunities for exposure of wildlife, domestic animals, and humans.

Interactions among wild and domestic hosts were also evident in the study by Lazár et al., who reported the first record of *Taenia lynciscapreoli* in Slovakia from a Eurasian lynx. In the same study, *Taenia krabbei* was identified in a gray wolf, golden jackal, and domestic dog, with genetic variation associated with different definitive hosts. These findings suggest that the transmission of *T. krabbei* may involve a broader network of carnivore hosts than traditionally recognized. In particular, free-ranging dogs and expanding golden jackal populations may interact with established sylvatic transmission cycles, further blurring the boundaries between wildlife and domestic parasite cycles.

The dynamic nature of host–parasite interactions was further demonstrated by Rau et al. Their report described the first documented case of severe pathological changes associated with *Cyathostoma americana* infection in a Harris's Hawk and, additionally, a Syngamidae–Aspergillus co-infection accompanied by intestinal endoparasitosis. This complex case illustrates that the outcome of parasitic infection cannot always be understood by considering a single parasite in isolation. Interactions among parasites, other infectious agents, host condition, and immune responses may collectively influence disease development and clinical outcome.

At a broader population level, Zhang et al. explored host–flea interactions in a plague-endemic region. Their findings demonstrated that both host species and individual host characteristics influence flea infestation patterns and identified *Spermophilus undulatus* as a particularly important species for future plague surveillance. The study emphasizes that vector abundance and distribution are closely linked to host ecology and that understanding these interactions is essential for designing effective surveillance strategies for vector-borne zoonoses.

Finally, Mohammed et al. provided a broader perspective on the technological advances that are transforming modern parasitology. Omics technologies, phylogenetics, structural biology, artificial intelligence, and immunoinformatics are increasingly used for antigen discovery, identification of drug targets, vaccine design, and epidemiological surveillance. These approaches offer unprecedented opportunities to explore host–parasite interactions at molecular and population levels. However, computational analyses remain constrained by incomplete datasets, uneven representation of parasite diversity, and simplified biological assumptions. Experimental validation therefore remains essential to ensure that computational predictions are biologically meaningful and applicable to naturally occurring host–parasite systems.

The studies included in this Research Topic collectively demonstrate the remarkable complexity and dynamic nature of interactions among parasites, wildlife hosts, domestic animals, vectors, and the environment. They show how parasites may adapt to new hosts, how expanding or invasive host populations can reshape parasite distribution, and how interactions between wildlife and domestic animals may influence established transmission cycles. At the same time, several contributions demonstrate that the epidemiological role of wildlife cannot simply be assumed from the detection of a parasite or pathogen; it must be interpreted in the context of host ecology, infection prevalence, transmission dynamics, and environmental conditions.

Advances in molecular diagnostics, phylogenetics, and emerging computational approaches are providing increasingly powerful tools to investigate these relationships. Nevertheless, detailed knowledge of parasite morphology, host ecology, pathology, and natural transmission cycles remains equally important. Integrating these approaches will be essential for understanding how host–parasite interactions respond to climate change, habitat modification, biological invasions, and increasing contact between wildlife, domestic animals, and humans.

This Research Topic therefore highlights a central message of modern wildlife parasitology: parasites and their hosts are part of continuously changing ecological and evolutionary interactions. Understanding these interactions is essential not only for documenting parasite diversity, but also for interpreting changes in wildlife health, recognizing emerging transmission patterns, and anticipating potential risks to animal and human populations.
